# Multilayer Dielectric Elastomer with Reconfigurable Electrodes for Artificial Muscle

**DOI:** 10.1002/advs.202206094

**Published:** 2023-01-19

**Authors:** Hongbo Fu, Yong Jiang, Jian Lv, Yao Huang, Zipeng Gai, Ying Liu, Pooi See Lee, Hong Xu, Daming Wu

**Affiliations:** ^1^ College of Mechanical and Electrical Engineering Beijing University of Chemical Technology Beijing 100029 China; ^2^ School of Materials Science and Engineering Nanyang Technological University Singapore 639798 Singapore; ^3^ Singapore‐HUJI Alliance for Research and Enterprise (SHARE) Smart Grippers for Soft Robotics (SGSR) Campus for Research Excellence and Technological Enterprise (CREATE) Singapore 138602 Singapore; ^4^ State Key Laboratory of Organic‐Inorganic Composites Beijing University of Chemical Technology Beijing 100029 China

**Keywords:** artificial muscles, detachable, dielectric elastomer actuator, electrode, multilayer structure

## Abstract

High‐performance multilayer dielectric elastomer actuators (DEAs) are well‐positioned to overcome the insufficient output force and energy density as artificial muscles. However, due to the fabrication process, the multilayer DEAs with nonmodifiable structures often suffer from the limitation of short lifespans and scalable preparation. Herein, reusable multilayer DEAs with the detachable and reconfigurable structure are fabricated. This is achieved by realizing scalable compliant electrodes using the continuous spatial confining forced network assembly (CSNA) method and combining the vacuum lamination (VL) approach to have good attachability and detachability with the VHB dielectric elastomer. The flexible roller‐based CSNA method is used to prepare the large area compliant electrodes composed of *α*, *ω*‐dihydroxy polydimethylsiloxane and electrically conductive nanoparticles. The fabricated electrodes can continuously work over 10 000 cycles at 40% strained stretching and maintain smooth surfaces to construct multilayer DEAs. Moreover, owing to the detachable configuration of the DEAs, the electrodes can also be recovered and reused for building new actuators. The lower limb assistive device is demonstrated by detachable multilayer spring roll DEAs, achieving approximately 3.1 degrees of flexion and extension movement of knee models under a voltage of 7 kV. The detachable and reconfigurable multilayer DEAs shed new light on the applications of wearable assistive devices.

## Graphical abstract

Multilayer dielectric elastomer actuators (DEAs) with detachable and reconfigurable capacity have been realized using the continuous spatial confining forced network assembly method and combining the vacuum lamination approach, offering new insight into the design of novel DEAs.

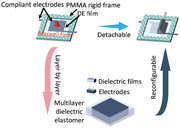

## 1 Introduction

Soft robots are advantageous for particular tasks of wearable rehabilitation devices,^[^ ^1^ ^]^ bio‐inspired actuators,^[^ ^2^ ^]^ and novel medical devices^[^ ^3^ ^]^ compared to traditional robotics with rigid structures and bulky control systems due to their lightweight, flexibility, and controllability.^[^ ^4^ ^]^ Among all components, artificial muscles play critical roles in the soft robotic system, which needs to provide considerable strain, sufficient energy density, and operating frequency.^[^ ^5^ ^]^ Electroactive polymers (EAPs) are promising stimuli‐responsive materials that are suitable for artificial muscles due to their reliable muscle‐like deformation under the effect of the electrical field.^[^ ^6^ ^]^ Among many potential EAP materials, dielectric elastomers (DE) have attracted much attention due to their sizeable electromechanical strain, high energy density, relatively fast response speed, lightweight, and silent operation.^[^ ^7^ ^]^ The standard DE devices consist of intermediate dielectric layers and two opposite electrodes. At present, a variety of DE materials are commercially available for dielectric elastomer actuators (DEAs), including natural rubber,^[^ ^8^, ^9^ ^]^ silicon rubber,^[^ ^10^, ^,11^ ^]^ polyurethane,^[^ ^12^ ^]^ acrylic elastomer (e.g., VHB 4905 and VHB 4910 from 3M company) et al. Common electrode materials include metallic paints (e.g., silver grease or nanowire), carbon grease, carbon nanoparticle or nanotubes, and conductive polymers. Nevertheless, the force output and displacement of single‐film dielectric elastomer devices remain challenging to satisfy the requirement of artificial muscles.^[^ ^13^ ^]^

The stacking structures have been shown to be able to generate more significant forces and displacements.^[^ ^14^ ^]^ Some efforts have been made to prepare multilayer devices by utilizing the acrylic elastomer.^[^ ^15^, ^16^ ^]^ However, the multilayer DEAs may also suffer from short lifespans due to the limitation of dielectric breakdown.^[^ ^17^ ^]^ Specifically, the dielectric breakdown is likely due to the unsuitable electric field and environmental factors^[^ ^18^ ^]^ or even the sharp tips of electrode‐induced corona discharging.^[^ ^19^ ^]^ Some failures are difficult to avoid due to the different fabrication processes. Thus, once localized damages occurred, the whole device could not be used again to accomplish the actuation process due to irremovable structures and unreusable electrodes. The electrodes in these works are hardly recovered from the sandwich‐structured actuators due to their bonding formed with the DE layer or non‐freestanding states. In comparison, the freestanding electrode delivering good attachability and detachability with dielectric elastomer is highly demanded to fabricate detachable and recyclable multilayer DEAs.

**Figure 1:**
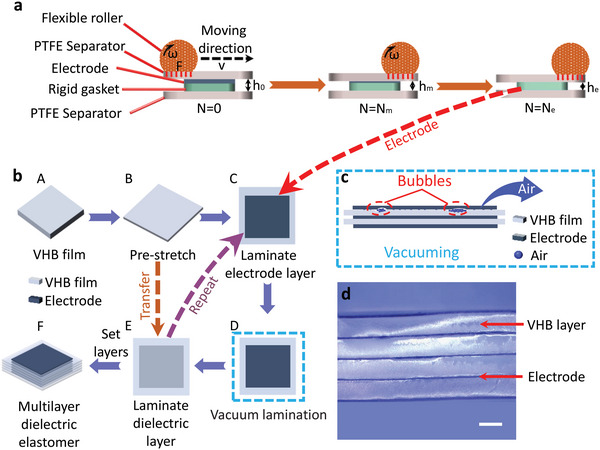
a) Schematic of CSNA method for the electrode of multilayer devices. b) Schematic of preparing process of multilayer devices. c) Schematic of the vacuum process for removing the bubbles. d) The optical cross‐section plot of the multilayer device with four layers VHB (Light‐grey layers) and five layers of electrodes (black layers) prepared via the vacuum lamination approach (without pre‐stretching).Scale bar, 1 mm.

Herein, we presented reconfigurable multilayer DEAs prepared via a new multilayer fabrication process and electrode material optimization to overcome the limitation of the nonreusable structure, toward the envisioned living artificial muscles for soft robotics. By the combination of CSNA^[^ ^20^ ^]^ and the vacuum lamination process, our approach enables detachable electrodes and modified multilayer DEAs. The detachable and reconfigurable structure benefited from the good attachability and detachability of electrodes prepared by the CSNA method and the favorable interlayer binding mode attributed to the vacuum lamination process. The schematic of the CSNA method based on the flexible roller is shown in **Figure** **1**a. This flexible roller‐based method provided a continuous compression process to prepare transferrable and recycled electrodes with suitable mechanical and electrical performance. The compliant electrodes exhibited a stretchability of 250% and cycling stability of at least 10 000 cycles at 40% uniaxial strain. The stacking process was to start with pre‐stretching VHB acrylic elastomer and the prepared electrodes were transferred to the surface. The whole device was moved to the vacuuming setup to remove the air bubble formed in the lamination process. Attaching a new DE layer to the top, laminating electrodes and the vacuuming process were repeated to complete the expected layers. The step‐by‐step fabrication process was presented in Figure 1b. With this approach, the spring roll actuators with 9 layers structure were prepared and successfully acted as the artificial muscles in a wearable lower limb assistive device. Intriguingly, the spring roll actuators have also been demonstrated to be detachable. This process opens up the opportunity to extend intelligently wearable devices comprised of multilayer DEAs with the help of detachable and reconfigurable structures.

## 2 Results and Discussion

Among elastomers for fabricating multilayer DEAs, acrylic elastomers possess a relatively high dielectric constant (4.5–4.8 at 1 kHz) and a theoretical energy density of 3.4 MJ m^−3^.^[^ ^21^ ^]^ They are also capable of withstanding relatively significant area changes (>380%).^[^ ^22^ ^]^ Because of their excellent properties, dielectric elastomers have great potential to be used as multilayer artificial muscle. Among them, VHB acrylic elastomer is a commercially available and frequently used material. Furthermore, due to internal crosslinking, acrylic elastomers have demonstrated excellent adhesion to other dielectric elastomers.^[^ ^23^ ^]^ When these elastomers are used as the dielectric layer, the total adhesive energy between separated DE layers and electrodes consisted of van der Waals force and intrinsic tackiness of acrylic layers.^[^ ^24^ ^]^ Such characteristics contributed to the good bonding with the electrodes constructed with even powder‐based electrodes, like carbon nanotube,^[^ ^25^, ^26^ ^]^ favoring the stability of the sandwiched actuators. Alternately, the surface roughness also influenced the interface energy.^[^ ^27^ ^]^ In contrast, the smooth surfaces of the electrodes benefited the efficient adhesion between layers.

In the present work, the electrodes were prepared by the CSNA method. The setup of the CSNA method consisted of a flexible roller, upper separator, lower separator, and rigid steel gasket. The polytetrafluoroethylene (PTFE) films were selected as the separators (refer to Figure 1a) due to their excellent chemical stability, high mechanical strength, stable superhydrophobicity, and smooth surface.^[^ ^28^, ^29^ ^]^ The chemical stability guaranteed that there is no chemical reaction with the electrode material. The high mechanical strength ensures no deformation during the compression process. The stable superhydrophobicity is responsible for the ease of removal of the electrode. The surface quality of electrodes fabricated by the CSNA method was guaranteed by the smooth top and bottom PTFE films. The surface of electrodes can be seen as the surface replication of PTFE films. This could improve the interlayer adhesive energy derived from the van der Walls forces.^[^ ^30^ ^]^ The combination of acrylic elastomer and compliant electrodes with smooth surfaces may pave the way to the reliability of multilayer DEAs.

The electrode material consisted of *α*, *ω*‐dihydroxy polydimethylsiloxane (107 silicone rubber), carbon black, carbon grease, ethyl orthosilicate, and dibutyltin dilaurate that were mixed together according to the suitable proportion in a homogenizer. The 107 silicone rubber was used as the binder, carbon black and carbon grease as the conductive agent, ethyl orthosilicate as the curing agent, and dibutyltin dilaurate as a catalyst. The mixture was transferred to the mold in the CSNA method, and the external force of 60 N was applied to make the flexible roller with the squeezing effect for the formation of a “plane‐to‐plane” compression area. The size of the different types of electrodes (one size was 40 mm long, 40 mm wide, and 120 µm thickness and the other was 120 mm long, 40 mm wide, and 120 µm thickness) can be co‐determined by the size of the gasket, the moving stroke of the flexible roller, the rolling number, and the applied force(F). After the compression process, the electrodes were peeled off the PTFE film to prepare for the subsequent tests and device fabrication steps.

Apart from the own membrane binding force, the air bubble formed during the preparation procedure may decrease the interlayer bonding energy. The air bubble may induce the non‐homogenous distribution of the electric field or even the breakdown of devices.^[^ ^31^ ^]^ Thus, the multilayer devices should be free of air bubbles. The schematic of removing air bubbles of multilayer devices by vacuum method was presented in Figure 1c, supporting overcoming these challenges and remodeling new devices. In practice, the multilayer device without pre‐stretching shown in Figure 1d was trimmed from the pre‐stretching device. The good adhesive states between electrodes and the dielectric layer and multilayer configuration are evidently shown.

### 2.1 Electrode Optimization

**Figure 2:**
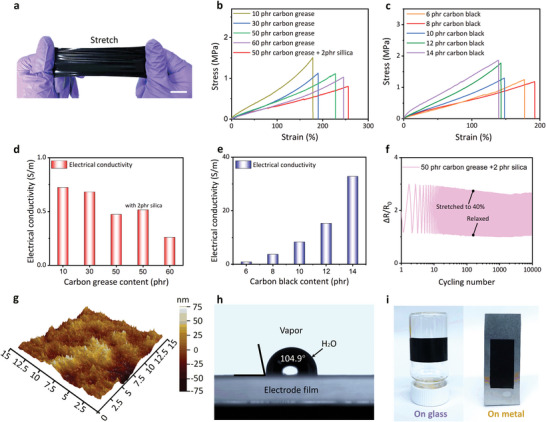
a) Optical image of the large‐scale fabricated electrode in the stretched state. Scale bar, 3 cm. b) Stress‐strain curves of electrodes filled with different content of carbon grease and silica (2 phr). c) Stress–strain curves of electrodes filled with different content of carbon black (without silica). d) Electrical conductivity versus the content of carbon grease. e) Electrical conductivity versus the content of carbon black. f) A cyclic test of the electrode under 40% strain. g) AFM image of the electrode film surface showing the roughness. h) Water droplet contact angle on the electrode surface. i) The adhesion of the electrode with curved glass and metal plate.

Due to the continuous preparation property of the CSNA method, the compliant electrode can be fabricated on a large scale according to the different occasions, as demonstrated in **Figure** **2**a. Moreover, the suitable mechanical and electrical performances of electrodes were beneficial for the actuation performance of DEAs. The electrical conductivity and mechanical properties may be achieved by densifying the conductive filler network formed by the “compression” effect of the flexible roller in the CSNA method. The tensile testing results of the electrode with different content of carbon grease was shown in Figure 2b. When the weight ratio of 107 silicone rubber and carbon black nanoparticles was fixed to 100 phr (parts per hundred parts of resin) and 5 phr, with the addition of carbon grease from 10 phr to 60 phr, it is clear that the elongation at break increased from 144.5% to 245.7%, respectively. At the same time, the tensile stress of the electrodes declined from 1.51 MPa to 1.02 MPa due to the silicone oil contained in carbon grease serving as the plasticizer to decrease the interaction of polymer chain and carbon black.^[^ ^32^ ^]^ In addition, a small amount of silica nanoparticles was introduced to improve the mechanical performance of electrodes, as it has been found to improve elongation at break^[^ ^33^ ^]^ and breakdown strength^[^ ^34^ ^]^ of composites. In comparison, the tensile strain lifted to 256.1%, and tensile stress declined to 0.8 MPa, which was because the silica nanoparticles can transmit the force to reduce slippage of the chain,^[^ ^35^ ^]^ and the “compression” effect of the CSNA method may compact the structure to amplify the improvement. It was also worth evaluating the relationship between the content of carbon black and the mechanical properties of the electrodes. The result in Figure 2c exhibits that the elongation at break and tensile strength changed from 192.4% and 1.1 MPa at 6 phr carbon black nanoparticles and 139.1% and 1.87 Mpa at 14 phr carbon black nanoparticles based on 100 phr 107 silicon rubber and 5 phr carbon grease. The stiffening of the matrix^[^ ^36^ ^]^ and the gradual forming of carbon black networks^[^ ^37^ ^]^ led to decreased tensile strain and increased tensile stress, respectively.

In addition to the mechanical properties, electrical conductivity is also an important parameter for the electrodes of DEAs, affecting the RC time constant of the actuator.^[^ ^38^ ^]^ As presented in Figure 2d, it was clear that the electrical conductivity of the electrodes gently declined with the increase of carbon grease content. The electrical conductivity of the electrodes dropped from 0.72 S/m to 0.26 S/m when the weight ratio of carbon grease increased from 10 phr to 60 phr, respectively, due to the non‐conductive grease preventing the formation of carbon black conductive networks in the electrodes.^[^ ^39^ ^]^ Notably, the introduction of silica caused no significant change in electrical conductivity. Figure 2e demonstrates the trends of electrical conductivity with the increasing amount of carbon black nanoparticles. It was apparent that the conductivity achieved 0.90 S/m and 32.8 S/m when the weight ratio of carbon black nanoparticles ranged from 6 phr to 14 phr due to the formation of a more efficient conductive network. The corresponding SEM images of the electrodes were added in Figure S1 (Supporting Information).

As for the electrodes of the actuator, the compliance and electrical conductivity^[^ ^40^ ^]^ were equally crucial to achieving good actuation performance. Alternatively, the carbon‐based electrode material works reasonably well, but drawbacks like rough surface were a barrier for multilayer devices.^[^ ^41^ ^]^ The electrodes prepared in this work were soft enough (elastic modulus<2 MPa), featured enough tensile strain (>130%), suitable electrical conductivity, and smooth surfaces, which could meet most of the electrodes requirement in building actuators.^[^ ^19^ ^]^ We mainly consider the tensile strain and tensile strength in the structure design of multilayer DEAs. Thus, we selected the electrodes consisting of 100 phr 107 silicon rubber, 50 phr carbon grease, 5 phr carbon black, and 2 phr silica for subsequent research. We also conducted other tests to evaluate the performance of this electrode. The working principle of dielectric elastomer is that the dielectric elastomer  can be deformed under induced Maxwell stress from the electrical field provided by the two opposite compliant electrodes.^[^ ^42^ ^]^ The compliant electrodes need to undergo electrostrictive deformation with the dielectric elastomer without losing conductivity. Therefore, we conducted the cycling test to evaluate the cycling resistance of electrodes to demonstrate the robustness of the electrodes. In our work, the maximum area strain of the single‐layer actuator based on the squares‐compliant electrodes was about 24%. That implied one of the side lengths may be elongated by around 12% in the lateral direction. To better ensure the reliability of the results, we test the cyclic electrical performance of the electrodes at the higher strain (40% strain) under the uniaxial stretching condition, as shown in Figure 2f. The stability of the relative resistance trend is retained for at least 10 000 cycles, which can satisfy the common deformation of actuators. The corresponding AFM image of the electrode surface was exhibited in Figure 2g. From this image, it can be seen that the average roughness Ra of the electrode was 12.55 nm, which was flat enough to decrease the inhomogeneity of the electric field. The contact angle of the electrode was 104.9° (Figure 2h) and displayed good adhesion with different materials, such as the glass container and aluminum plate (Figure 2i), which enabled the heliport to improve the interlayer binding force in the multilayer DEAs. Cumulatively, these properties confirmed the potential of electrodes in constructing multilayer DEAs for artificial muscles.

### 2.2 Detachable and Reconfigurable Capability

**Figure 3:**
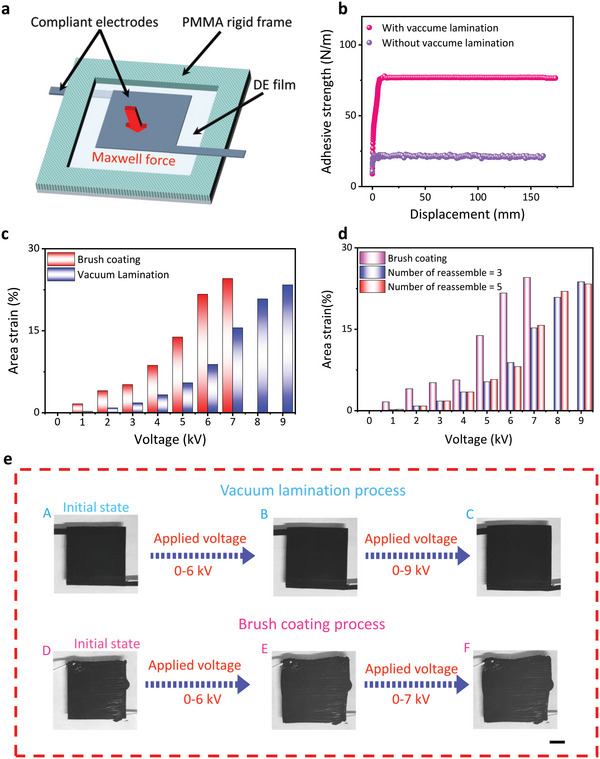
a) The actuation mechanism of the dielectric elastomer under electrostatic force. b) Adhesive strength (peel test at 100 mm min^−1^) between VHB and laminated electrode with or without vacuum lamination approach. c) The comparison of the electrically induced actuation strain under the electrodes prepared through the traditional brush coating process and vacuum lamination approach. d) The actuated strain of the actuator versus the number of recycling electrodes (The 370% equibiaxially pre‐stretched VHB was applied to voltage via the recycled electrodes prepared by the vacuum lamination approach and brush coating process). e) Captured optical images from a video. A before actuation, B at 6 kV, and C at 9 kV. The electrodes in A‐C were fabricated by the vacuum lamination process. D before actuation, E at 6 kV, and F at 7 kV. The electrodes in D‐E were fabricated by the brush coating process. Scale bars, 1 cm.

To compare the difference between the electrodes fabricated through the vacuum lamination and the traditional brush coating process in the application of the electrically actuated field, the actuation performance of DEAs with one single dielectric layer was used as an evaluation method. The actuation mechanism of DEA was shown in **Figure** **3**a. The dielectric films displayed electrostrictive performance under the effect of Maxwell force based on the electrical field provided by the two compliant electrodes (positive and negative sides).^[^ ^43^ ^]^ Of course, the interlayer binding forces are also crucial for the actuation performance. Thus, to accurately evaluate the adhesion performance of the electrode and VHB layer in the vacuum lamination process, a 180° peeling test was used to measure the adhesion force (see Movie S1, Supporting Information). The relationship between adhesive strength and displacement of the device was shown in Figure 3b. Owing to the intrinsic adhesion of the VHB films and the effect of van der Waals forces of interlamination, the surface adhesive force per length can reach up to 76.5 N m^−1^, which contributed to the prevention of slipping and peeling off of the electrodes from the surface of VHB layers. As shown in Figure S2a (Supporting Information), the electrostrictive movement of dielectric elastomer actuators with sandwich‐like structures can be splited into the active deformation of the dielectric elastomer under Maxwell force and then be delivered to the passive deformation of compliant electrodes through the contact area (yellow area). When the electrostrictive coefficients were neglected, the lateral output stresses (*σ*_x_ and *σ*_y_) of a single dielectric elastomer layer actuator were calculated according to Equation S1 (Supporting Information).^[^ ^44^ ^]^ The adhesive force between the electrodes and dielectric elastomer is mainly derived from the electric field‐induced friction stress (*p*_s_) and interlayer bonding force from the material during the electrostrictive movement (shown in Figure S2b, Supporting Information). According to the calculated equation of Maxwell force, the frictional shear stress can be calculated via Equation S2 (Supporting Information). The acrylic dielectric elastomer and the rubber electrodes are not low‐friction membrane materials,^[^ ^45^ ^]^ the friction coefficient is >1. Thus, the frictional shear stress between the electrode and dielectric elastomer is larger than the lateral output stress of the dielectric elastomer, which implies that the interfacial fraction force could satisfy the requirement of transferring the output force of the dielectric elastomer. Additionally, the peel test has proven that there is a certain interlayer bonding force. Combining the frictional force and interlayer bonding force, the interlayer slippage and peel between electrodes and dielectric elastomer can be avoided. In comparison, for the lamination without the vacuum process, the peeling strength had only 21.67 N m^−1^, which demonstrated the effectiveness of the vacuum process in improving the interlayer binding force for facilitating the stable DEAs with multilayer structure. However, it should be noted that, for the brush coating process, electrodes were hard to peel off from the VHB layer in this work, which may be due to the different binding modes provided by the brush coating process. Another interesting feature of this vacuum lamination approach was its detachable property, which permitted the repeated use of electrodes and even replaced specific layers to extend the lifespan of the device. Figure S3 (Supporting Information) demonstrated the disassembly procedure of the single‐layer DEA. Firstly, due to the certain mechanical strength, the electrodes can be gently peeled off by the tweezers along their outer edge (step A). Then the stripped electrodes were adhered to the glass rod (step B) and turned around, following the glass rod to peel from the surface of the VHB film (step C). The electrodes were collected in the container by turning around the glass rod in the reverse direction (steps D and E). The opposite can also be disassembled by repeating the above process. After disassembling electrodes at both ends, the VHB layer can be disassembled or substituted from the frame. Figure 3c demonstrates the relationship of actuation strain of sandwiched DEAs (including one 370% equibiaxially pre‐stretched VHB layer and two compliant electrodes) under the electrical field provided by the electrodes prepared by the brush coating process and vacuum lamination approach. The breakdown voltages applied to the VHB layer were 7 kV and 9 kV for the two types of electrode, respectively, considering the stable dielectric strength of VHB, which was probably determined by the distinguishable spacing between the dielectric layer and electrode caused by the different processes. Moreover, the actuation strains of the two samples were significantly raised to 24.55% and 23.37%, respectively, which were limited to the area of this actuator and their mechanical performance. Specifically, for the dielectric elastomer, the contracted thickness can be calculated according to Equation S3 (Supporting Information).^[^ ^46^ ^]^ The area strain of the dielectric elastomer can be estimated by the incompressible principle of the dielectric elastomer. However, the dielectric elastomer needed to move together with compliant electrodes on the two ends, which would have an effect on the area strain of the VHB dielectric elastomer. The robust composite electrodes allowed low mechanical loss and good adhesion to the dielectric elastomer,^[^ ^47^ ^]^ but the incorporation of conductive filler and the elastomer itself increased the stiffness of the actuator, which was the main reason for the lower actuation performance. This phenomenon is in agreement with the reports in the literature.^[^ ^48^, ^49^, ^50^ ^]^ Obviously, adhesion between VHB and electrode layers in these two processes allowed their simultaneous movement (stretch or compress). Figure 3d shows the comparison of the area strain of sandwiched actuator consisting of 370% equibiaxially pre‐stretched VHB film and recycled electrodes at different times of use. The recycled three times and the recycled five times electrodes underwent 23.14% and 23.87% area change at a voltage of 7 kV, respectively, demonstrating the recyclability of electrodes in the actuators prepared via the vacuum lamination process. Figure 3e presents one group of corresponding photographs related to area strain in different voltages of the actuators in Figure 3c. As was evident from Figure 3e, the electrodes prepared by the CSNA method have a smoother surface than the electrode fabricated by the brush coating process. In addition, the integrated electrodes were easier to prepare in this process, which may avoid the tip discharge in the transfer regions formed by the flexible electrodes and rigid copper wires.^[^ ^51^ ^]^ Generally, these tests provided the primary platform for designing different actuators to meet various application fields. To validate the structural stability of the sandwiched actuator prepared in this work. The cyclic actuation test of 370% equibiaxially pre‐stretched VHB 4910 was successfully performed at 1 Hz frequency. As presented in Figure S4 (Supporting Information), the *z*‐axis displacement first increased gradually and then progressively declined with the increase of cyclic numbers, which may be attributed to the viscoelastic effect of the VHB layer. Moreover, the response of the single‐layer actuator at 1, 10, 100, and 1000 Hz frequencies was shown in Movie S2 (Supporting Information), which demonstrated the operation of the single‐layer actuator at high frequencies.

### 2.3 Artificial Muscle Demonstrations

**Figure 4:**
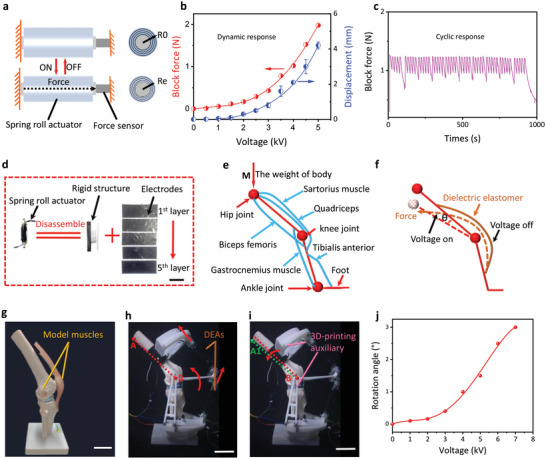
a) Schematic of the testing method of block force. b) The force‐displacement‐voltage behavior of the spring roll actuator based on multilayer devices (consisting of five layers electrodes and four layers VHB layer with 370% equibiaxially pre‐stretched). c) The cyclic test of the spring roll actuator under the voltage range from 0 V to 4 kV. d) Recycling electrodes from the spring roll actuator. e) Schematic of lower limb muscles. f) Schematic of the actuation mechanism of DEAs assistive devices. g) The model of the human muscle around the knee. (h) The installation position of DEAs and relevant 3D‐printing auxiliary on the model. i)The working state of lower limb assistive devices. j) The rotation angle of the model versus the increase of applied voltage. All scale bars, 5 cm.

DEAs have many applications in soft robotics, like the soft gripper, locomotion robots, and artificial muscles, due to their compliance and flexibility, similar to natural creatures.^[^ ^52^ ^]^ The spring roll actuator showed its simplicity and multifunctional properties as an artificial muscle.^[^ ^53^ ^]^ To validate the stability and reliability of multilayer devices prepared by our approach. We fabricated the spring roll actuators based on the detachable multilayer devices. The block force and free displacement in the axial direction of the spring roll actuator with a nine layers structure were measured. **Figure** **4**a presents the testing principle of the block force produced by the spring roll actuator. The spring roll actuator produced the force and displacement when DE films were activated by the input voltage due to the net strain on the DE films and the spring.^[^ ^14^ ^]^ The relationship between the applied voltage and dynamic response (block force and displacement) is plotted in Figure 4b. When the applied voltage rose from 0 V to 5 kV, the block force and free displacement gradually increased to 1.98 N and 4.2 mm, respectively, demonstrating the reliability of multilayer structures. To test the stability of the actuator, the loop voltage applied from 0 V to 4 kV voltage cycles with 0.2 Hz frequency was repeated for 50 cycles. The experimental result is presented in Figure 4c. The approximate extent of the block force varied within the interval of 0.8 N to 1.2 N under voltages ranging from 0 V to 4 kV. The trend of block force is due to the strong viscoelasticity of VHB film, which resulted in a long time taken to recover from the initial strain.^[^ ^54^ ^]^ However, since we have added a high actuation frequency, the deformation of the actuator could not return to zero. As shown in Figure 4c, the residual stress remained even after about 60 s (940 s – 1000 s). The viscoelasticity of the VHB layer also contributed to the lower response speed of the actuator. Moreover, the cyclic testing further demonstrated the stability of the structure. There were many reasons that caused the failure of dielectric elastomers as shown in the literature,^[^ ^55^, ^56^ ^]^ which limited the extensive application of DEAs. Some ways have been used to detect the different breakdowns of DEAs,^[^ ^57^, ^58^, ^59^ ^]^ which may be further applied to detect the failure of the DE layers in multilayer. Once the damaged layers may be replaced, the electrode layer in the multilayer structure fabricated by the vacuum lamination process may be recycled to form the new device. Figure 4d shows the recovered electrodes from the spring roll actuator when the VHB layers were disrupted, which provided novel ideas for detachable and reconfigurable actuators. Linear DEAs like roll actuators (with or without spring) and stacking actuators were practical structures to convert the output force direction of extension of dielectric elastomer into axial expansion for the application of artificial muscles.^[^ ^60^ ^]^ The mechanical work output (*E*_mech_ = *F*_b_ × *D*, where *E*_mech_ represented the output mechanical energy, *F*_b_ represented the block force, and D represented the displacement) of the linear actuator was a significant parameter that affected the use of the actuators.^[^ ^13^ ^]^ Figure S5 (Supporting Information) demonstrates the output mechanical energy comparison between the previously reported linear DEAs based on multilayer configuration^[^ ^13^, ^16^, ^38^, ^61^, ^62^, ^63^ ^]^ and the spring roll actuator prepared in this work. The output mechanical energy of the spring roll actuator was mainly derived from the contribution of the multilayer DEAs prepared in this work and the spring. Moreover, the detachable and reconfigurable capacity of the multilayer DEAs can contribute to the development of linear DEAs. Overall, we have demonstrated the practicability of stacking multilayer configurations for preparing DEAs based on our approach, expanding the potential applications in other detachable devices.

Among the numerous application fields of DEAs, the frequently reported incidence of stroke worldwide is a subject of intense scrutiny.^[^ ^64^ ^]^ Patients often suffered physical inactivity after stroke due to losing muscle mass, strength, and function.^[^ ^65^, ^66^ ^]^ A proper physical rehabilitation like knee extension‐flexion and walking test may promote brain and muscle function recovery.^[^ ^67^ ^]^ However, there are shortcomings of bulkiness and challenging to implement multimodal control.^[^ ^68^, ^69^ ^]^ The dielectric elastomers may overcome deficiencies by relying on their combined advantages resembling natural muscles,^[^ ^42^ ^]^ and modified multilayer DEAs may further promote the development. To explore the potential application of DEAs in lower limb assistive devices, we designed assistive equipment based on the distribution of lower limb muscles for bending gait. Figure 4e presents the schematic of human lower limb muscle systems. Naturally, every movement of the lower limbs required muscle cooperation.^[^ ^70^ ^]^ For example, when we are moving, the muscles need to not only push the body but also keep the balance with the help of other muscles. Figure 4f exhibits the possible actuation mechanism of the assistive devices based on DEAs. When the voltage was applied, the force and displacement of actuators were along the axial direction. Thus, the 3D‐printed auxiliary should be used to change the moving direction of the lower limb. When the voltage was revoked, the resilience of the elastomer will drive the lower limbs to the initial position. The bending angle was defined as *θ*. In order to explore the feasibility of this idea, we designed the devices on the lower limbs model, as presented in Figure 4g. The model demonstrated the combination of muscles around the knee joint. We used the spring roll actuator to replace the “muscle” on the model to accomplish the knee flex and extension task. Figure 4h displays the original installation of the devices, including the DEAs and related attachments. When voltages were applied to the actuators, the actuator output the force (red arrow), and then the model bent. Due to the resilience of the elastomer, the model returned to point A when the voltage was cut off (see Movie S3, Supporting Information). The output direction of the spring roll actuator was changed via rotatable pivot B. Figure 4i shows the moving state of lower limbs with the help of the assistive device. The bent from point A (red line) to A1 (green line). The correspondence of voltage and rotation angle *θ* of the model was demonstrated in Figure 4j. The rotation angle increased to ≈3.1 degrees when the voltage was ramped to 7 kV, suggesting the feasibility of the program.

Definitely, the devices were insufficient to complete most of the movement of lower limbs, which was limited by the output performance of DEAs and the stretchability of the original “muscles” on the model. Additionally, each movement of the lower limbs required mutual coordination of lower‐limb muscle systems. A lower limb system based on DEAs should be prepared for multimodal movement of the lower limb in future work, which may be used as convenient wearable devices for the rehabilitation training of stroke patients due to the advantages of DEAs. In general, the detachable and reconfigurable multilayer DEAs paved the way for the further development of lower limb assistive devices.

## 3 Conclusion

We created multilayer DEAs with detachable and reconfigured structures for artificial muscles via the combinatorial process of the CSNA method and vacuum lamination approach. The CSNA method provided the compliant electrodes with suitable actuation performance and smooth surfaces for the multilayer DEAs. The vacuum lamination method promoted the interlayer binding force and homogeneous distribution of electrical field for the multilayer DEAs by removing the air bubbles between VHB dielectric layers and electrodes. The significance of this work lies in the combination of the material optimization and forming process enabling the DEAs with detachable structures. The electrodes could be recycled from the DEAs and reutilized to fabricate new devices. The spring roll actuator and a wearable lower limb assistive device based on the multilayer dielectric elastomers demonstrated the potential application of this process. Moreover, thin and compliant electrodes of different sizes can be prepared through the continuous CSNA method for different scenarios. The combinatorial approach of separated DE layers and electrodes indicated a novel process for creating multilayer DEAs. Beyond stacking for improving the actuation performance of DEAs, our approach is promising for a wide range of applications in detachable sensors and intelligently wearable devices for rehabilitation.

## 4 Experimental Section

### Material

The commercially VHB 4910 acrylic elastomer films (initial thickness, 1 mm) were purchased from the 3M corporation. The polymeric matrix for the electrodes 107 silicon rubber was supplied by Shenzhen Hongyejie Technology Co., Ltd, which could be cured at room temperature. Ethyl orthosilicate (Beijing Tongguang Fine Chemical Co., Ltd. China) was the curing agent. Dibutyltin dilaurate (acting as the catalyst) was purchased from Shanghai Maclean Biochemical Technology Co., Ltd, China. The carbon grease (Yamate, GV‐80s) was supplied by Suzhou Zhengfengyi Lubricating Material Co., Ltd, China. The conductive carbon grease (GV‐80s) and the Ketjenblack ECP‐600JD were conductive fillers. Vapor‐phased silica was produced by Degussa Company. The flexible roller in the CSNA method, with a length of 72 mm and a diameter of 66 mm, was provided by Yatong Environmental Protection Machinery Co., Ltd., China. The mold, namely Poly(tetrafluoroethylene (PTFE) film with 0.2 mm thickness, was purchased from Shenzhen Huasheng Plastic Materials Co., Ltd, China. The rigid gaskets were provided by Curing Stainless Steel Co., Ltd, China.

### Preparation of Freestanding Electrode Thin Film

The mixed ratio of 107 silicon rubber(100 phr), ethyl orthosilicate (20 phr), and dibutyltin dilaurate (12 phr) mixture were fixed. This was due to the reduced curing time with the increase of dibutyltin dilaurate. In particular, the curing time was 1.5 h at room temperature when the weight ratio of dibutyltin dilaurate achieved 12 phr, favoring the increase in efficiency. In order to guarantee the mechanical property of the electrode, the curing time was determined to be 12 h as the mixture was enclosed in the mold. Two corresponding formulations were investigated to evaluate the effect of different conductive fillers. The first one: carbon grease was added from 10 to 60 phr when the weight ratio of carbon black was fixed to 5 phr. The second one has a weight ratio of carbon black nanoparticles changing from 6 to 14 phr when the content of carbon grease was fixed to 50 phr. The blend of 107 silicon rubber, Ethyl orthosilicate, Dibutyltin dilaurate, carbon grease, carbon black nanoparticles, and silica nanoparticles was mixed 2 min at 1800 rpm in a homogenizer from Shenzhen Zhongyi Technology corporation. The mixture was transferred to the mold of the CSNA method, which consisted of three structures (upper PTFE film, rigid gasket, and lower PTFE film). The external force (vertical direction) of 60 N was applied at a flexible roller, and the size of the ″rectangular plane formed by compression was length: 72 mm × width: 40 mm. The moving speed of the flexible roller was controlled to 5 mm s^−1^. The “plane to plane” region was delivered with the help of horizontal force. The number of the rolling cycle was fixed to 20. After the entire process (rolling number from *N*_0_ to *N*_e_ and the thickness from *h*_0_ to *h*_e_), the whole mold was placed in the air for curing, and the composites were peeled off the mold for the following process. The thicknesses of electrodes were controlled to around 120 µm through the declined distance of the flexible roller and the thickness of the rigid gasket.

### Fabrication of the Multilayer Devices

The electrodes were first prepared through the CSNA method and peeled off the mold. Then, the device was made through the vacuum lamination process layer by layer as shown in Figure 1b. First, the VHB acrylic elastomers were fixed to the PMMA rigid frame with the equibiaxial pre‐stretch of 370% × 370% (step B). The electrode (length: 120 mm × width: 50 mm) was transferred to the surface of the VHB acrylic elastomer with the assistance of a round rod (step C). The whole device was transferred to a vacuum freeze dryer (Ningbo Shuang Jia Instrument Co. Ltd.) as the vacuum degree was 1000 Pa for 2 h at room temperature to remove the bubbles and air formed during the stacking layer or reconfigure the structure between layers. The pre‐stretch, electrode attachment, and vacuum lamination process (steps B, C, D, E) were repeated to prepare the specific multilayer devices.

### Fabrication of the Spring Roll Actuator

The spring roll actuator usually was composed of the center spring and stretched dielectric devices (including elastomers and electrodes). The spring was precompressed, and the stretched multilayer device was transferred to the spring under the pre‐stretched condition. Two end caps should be added to maintain the structure. In this work, the inner and outer diameters were 8.5 mm and 12.5 mm, respectively. The spring constant was 0.62 N mm^−1^, which was determined by the modulus of the DEA. All electrode lengths were 120 mm with a width of 25 mm. High voltage and ground should be applied via conductive wires. The schematic and photographs were shown in Figure S6a,b (Supporting Information). The activated interval was the location at which the electric field was applied, overlapping the spring (5–7 cm between compressed and relaxed states). High voltage was applied to the actuators via the DW‐P153‐50AC‐2 (Tianjin Dongwen high voltage supply factory, China). The block force was measured through the YLK‐2 thrust gauge on the free side of the actuator. The displacement of the actuator was recorded via the camera, and the images were processed.

### Fabrication of the Lower Limb Assistive Device

To explore the possibility of wearable lower limb assistive equipment based on multilayer devices. A set of 3D printing components and relative accessories were fixed to the 3D musculoskeletal knee model of the lower limb, and the spring roll actuators were used to be the artificial muscle for replacing the function of the muscles to help the movement of flexing the knee. Due to the motion of the spring roll actuator along the axial direction, a turning structure was designed to change the moving direction.

### Characterization of the Electrodes

Scanning Electron Microscope (SEM) micrographs of the dispersion of fillers in the polymer matrix were obtained via the Hitachi S‐4700 and JEOL scanning electron microscope (JED‐2300). The measurements were conducted on the uniaxial tension mode using mechanical tester MTS C42 with a loading speed of 20 mm min^−1^, following ASTM D638. The size of the samples was prepared according to ASTM D638 type V. The electrical conductivities were measured using a standard four‐probe station (HPS2524). The Uniaxially cyclic tests were conducted with 40% strain at 20 mm min^−1^ tensile rates via the Mark‐10 ESM303 Motorized Test Stand. The experimental result of resistance was recorded via a data acquisition system (including a DAQ6510 data acquisition system and KickStart Instrument Control Software). The relative change in resistance was defined as Δ*R*/*R*_0_, where *R*_0_ and Δ*R* are the electrical resistance of the electrode without and with deformation, respectively. Atomic force microscope (AFM) images were recorded on NX10 AFM. The Data physics OCA15EC (Data Physics Instruments GmbH) detected the static contact angle. All of the tests were performed at room temperature.

### Peel Test

The T‐peel test was used to measure the adhesive force between the electrode layer and VHB layer with a peeling angle of 180° according to the GB/T 2792‐2014 on CMT4503. The device was prepared in the same process as multilayer devices. The VHB layer was fixed to the polycarbonate (PC) substrate at 0.4 mm to guarantee the same stretching rates. The width of the electrode was 25 mm with a length of 125 mm, and the peeling length was 100 mm. The peeling speed was 100 mm min^−1^ at room temperature. The forces were recorded by a force transducer. The peel strength was calculated via the equation:$\sigma_{P}=\frac{F}{B}$, where *σ*_*P*_ represented the peel strength, *F* was the peel force, and B was the width of the sample. The experiments were replicated three times, and the results were expressed as the mean value. The contrast tests were prepared using the same process without the vacuum process.

### Actuation Properties of the Actuator with a Single Dielectric Layer

For the test of the actuation properties, the VHB 4910 films with a thickness of 1 mm were fixed to the PMMA rigid frame with 80 mm × 80 mm square in the inner portion. The original size of the electrode prepared by the CSNA method and applicator brush coating method was the same 40 mm in length and 40 mm in width. It should be noted that the thickness of the electrode prepared by the brush coating method was near 230 µm due to the limitation of molding process. The preparation process for single‐layer devices was the same as the above process for multilayer devices. The cross‐section photograph of the single‐layer devices (without pre‐stretching) was presented in Figure S7 (Supporting Information). The high voltage was provided via the high voltage source, and a digital camera recorded the actuation process. The actuation strain was tabulated through the MATLAB program based on the grayscale conversion. As for the recycling test of the electrodes, the process was kept consistent with the above process.

### Cyclic Test

The equibiaxial prestretch of 370% × 370% VHB 4910 was fixed to the rigid frame with 60 mm × 60 mm square in the inner portion. The electrode was 20 mm wide, 20 mm in length, and has a thickness of 120 µm. The square waveform was controlled to 5 kV at a frequency of 1 Hz by TTi TGP110 10 MHz Pulse generator and delivered to the output high voltage amplifier (Trek Model 610E, Trek Inc.). A laser displacement sensor (optoNCDT ILD2300) was used to record the vertical displacement (*z*‐axis) in the center of the actuator at 2.5 kHz frequency.

### Frequency Response

The sample size and fixed frame were the same as those used in the cyclic test except for the frequency value.

### Characterization of the Spring Roll Actuator with Multilayer DEAs

To test the dynamic response of the spring roll actuator, the experimental process required the application of different voltages to the spring roll actuator from 1 kV to 7 kV. The block force and the stroke of the free side were recorded. In addition, to verify the cycling response of the spring roll actuator, considering the dielectric strength and viscoelastic property, the dynamic properties of the spring roll actuator were cycled 50 times under the voltage cycle from 0 V to 4 kV with a 5‐second (s) cycling period.

### Characterization of the Lower Limb Assistive Devices

The four actuators were applied to the different bias voltages of 0–1, 0–2, 0–3, 0–4, 0–5, 0–6, and 0–7 kV, respectively. A camera recorded the moving angles and strokes of the lower limb model. The results of the rotation angle were calculated through the images.

## Conflict of Interest

The authors declare no conflict of interest.

## Supporting information

Supporting Information

Supplemental Movie 1

Supplemental Movie 2

Supplemental Movie 3

## Data Availability

The data that support the findings of this study are available from the corresponding author upon reasonable request.
