# Supplementary material for: Multilayer Dielectric Elastomer with Reconfigurable Electrodes for Artificial Muscle
Source: Adv Sci (Weinh). 2023 Jan 19;10(9):2206094. doi: 10.1002/advs.202206094 (PMC10037966; doi:10.1002/advs.202206094)
Supplement: Supplementary file 1 — Supporting Information [file ADVS-10-2206094-s001.pdf]

Multi-layer Dielectric Elastomer with Reconfigurable Electrodes for Artificial Muscle

Hongbo Fu[a], Yong Jiang[a], Jian Lv[b], Yao Huang[a], Zipeng Gai[a], Ying Liu[a], Pooi See Lee[b, c]*, Hong Xu[a,d]*, Daming Wu[a,d]*

a. College of Mechanical and Electrical Engineering, Beijing University of Chemical Technology, Beijing 100029, China.

b. School of Materials Science and Engineering, Nanyang Technological University, Singapore 639798, Singapore.

c. Singapore-HUJI Alliance for Research and Enterprise (SHARE), Smart Grippers for Soft Robotics (SGSR), Campus for Research Excellence and Technological Enterprise (CREATE), Singapore 138602, Singapore.

d. State Key Laboratory of Organic-Inorganic Composites, Beijing University of Chemical Technology, Beijing 100029, China.

*Corresponding emails: pslee@ntu.edu.sg; xuhong@mail.buct.edu.cn; wudaming@vip.163.com.

To further explore the mechanism behind the mechanical and electrical performance of the compliant electrodes. We used the cross-section SEM images to illustrate the dispersion in the polymer matrix. Figure S1a-e presented that the carbon blacks and carbon greases were uniformly dispersed in the polymeric matrix, indicating that the CSNA method can help build the electrically conductive network. One can see that the content of carbon black increased gradually with the content of carbon grease. However, the grease content was also increased, which was one of the main reasons for the decline of the tensile stress and electrical conductivity of the electrodes. It was consistent with the results in Figure 2b and Figure 2d. The morphological difference of electrodes with the increasing carbon black content can be found through SEM images, as shown in Figure S1f-i. The SEM showed a change in the carbon black network, underlying cause of improving electrical conductivity, elongation at break and tensile stress, as presented in Figure 2c and Figure 2e.

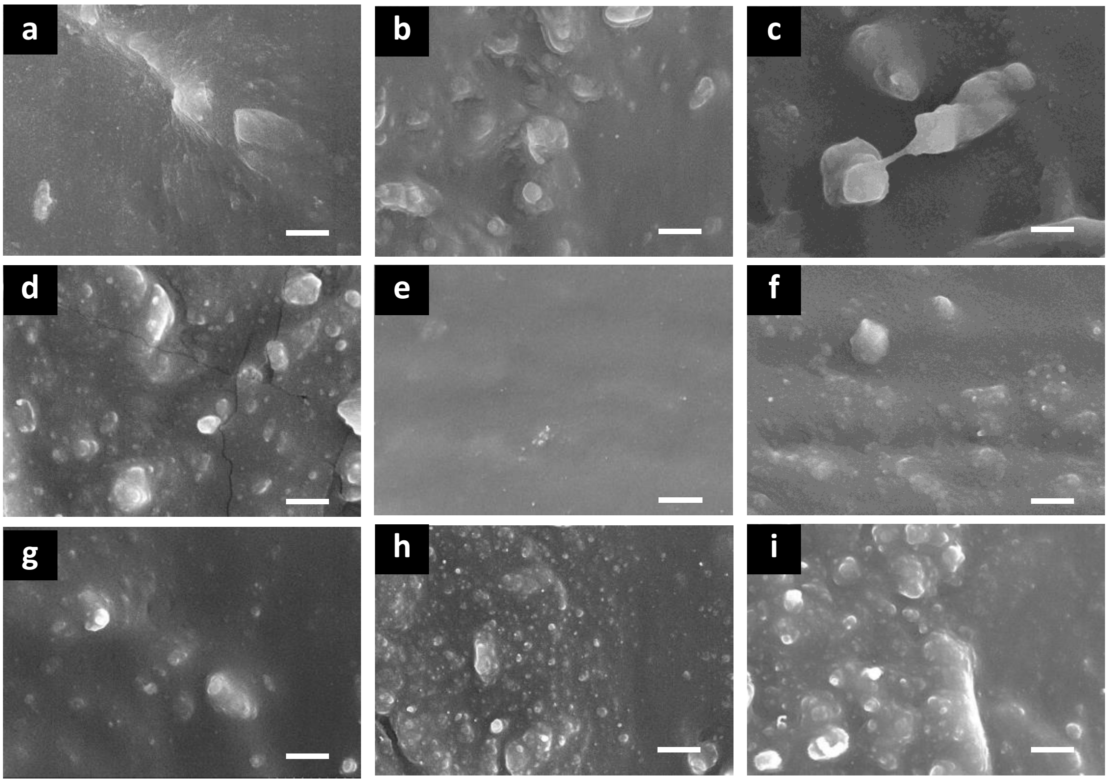

Figure S1. The cross-section SEM images of electrodes. (a) 10 phr carbon grease, (b) 30 phr carbon grease, (c) 50 phr carbon grease, (d) 60 phr carbon grease, (e) 50 phr carbon grease and 2 phr silica. (a)-(e) with the same concentration of other filler, including 100phr 107 silicon rubber /5 phr carbon black. (f) 6 phr carbon black, (g) 8 phr carbon black, (h) 10 phr carbon black, (i) 12 phr carbon black. (f)-(i) with the same concentration of other filler, including 100 phr 107 silicon rubber /50 phr carbon grease. All scale bar, 5 μm

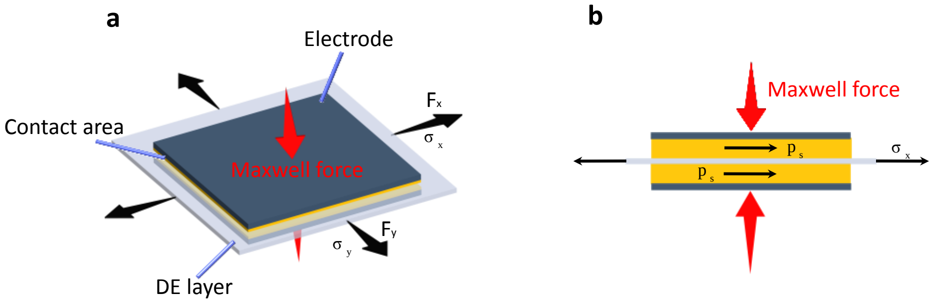

Figure S2. (a) Schematic of stress and force distribution on the dielectric elastomer actuator with the single layer dielectric elastomer. (b) Schematic of interlayer stress distribution in single-layer dielectric elastomer actuator. (Yellow region represented the contact area of electrodes and dielectric elastomer)

$$\sigma_x = \sigma_y = \frac{1}{2}\varepsilon_0\varepsilon_r E^2 \tag{S1}$$

Where σ_x was the stress in the x-direction, σ_y was the stress in the y-direction, ε_0 was the permittivity of vacuum (ε_0 =8.8542 × 10 $^{-12}$ F/m), ε_r was the relative permittivity, and E was the electric field strength.

$$p_s = \frac{1}{2}\mu\varepsilon_0\varepsilon_r E^2 \tag{S2}$$

Where p_s was the friction shear stress, μ was the friction coefficient. ε_0 was the permittivity of vacuum (ε_0 =8.8542 × 10 $^{-12}$ F/m), ε_r was the relative permittivity, and E was the electric field strength.

For the dielectric elastomer, the contracted thickness can be calculated according to the following equation：

$$S_t = -P / Y = -\varepsilon_0\varepsilon_r E^2 / Y \tag{S3}$$

Where S_t represented the decrease in thickness, P was the Maxwell force, Y was the elastic modulus of dielectric elastomer and electrodes, ε_0 was the permittivity of vacuum (ε_0 =8.8542 × 10 $^{-12}$ F/m), ε_r was the relative permittivity, and E was the electric field strength.

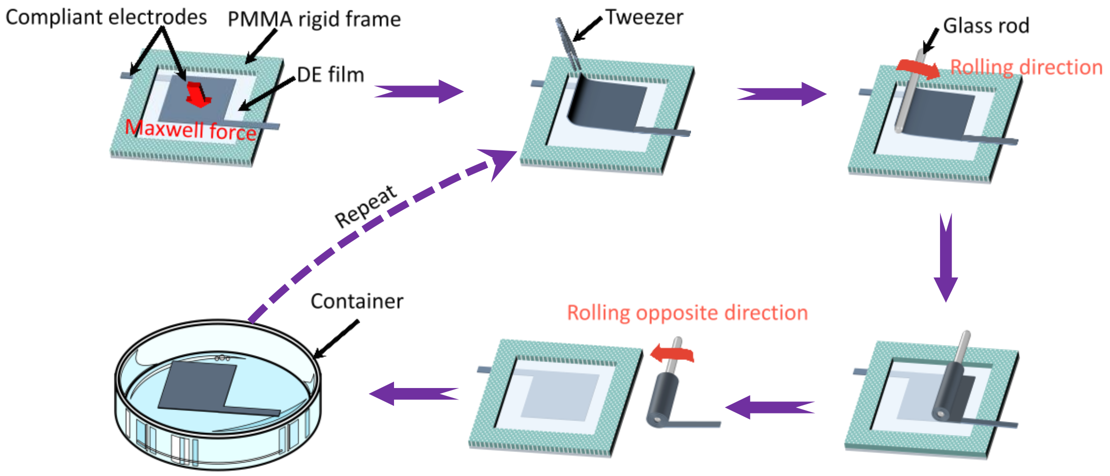

Figure S3. Schematic of the disassembly process of single-layer dielectric elastomer actuator.

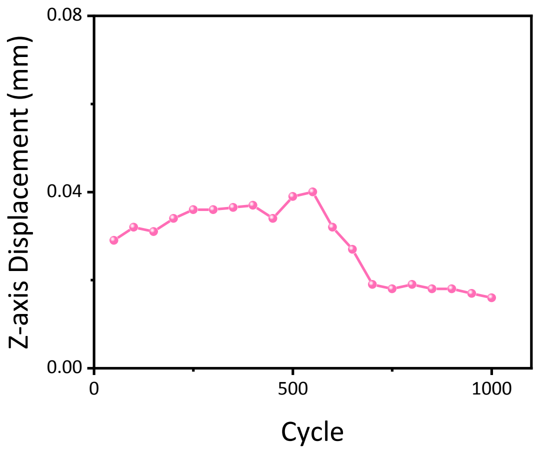

Figure S4. Cyclic actuation test of 1000 cycles on 370% equibiaxially pre-stretched VHB^{TM} 4910 at the applied voltage of 5 kV and the excitation frequency of 1Hz.

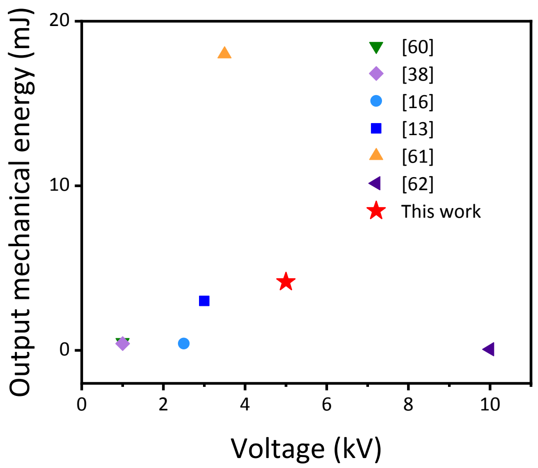

Figure S5. The comparison with the output mechanical energy of our spring roll actuator based on the multilayer DEAs and previously reported linear actuators.

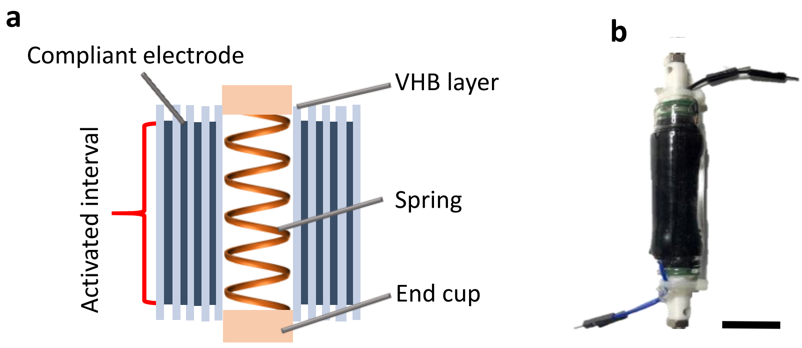

Figure S6. (a) The schematic structure of the spring roll actuator with the multilayer devices. (b) The photograph of the sample of the spring roll actuator. Scale bars, 20 cm.

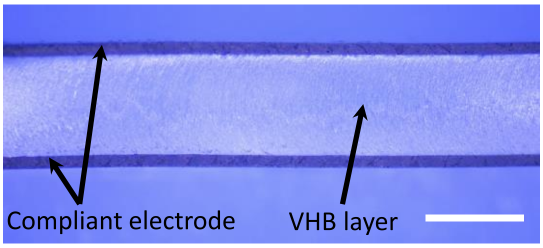

Figure S7. The cross-section plot of the sandwiched device with layer VHB and two opposite electrodes based on the vacuum lamination approach (without prestretch). Scale bars, 1cm.
